# Angiosome-directed endovascular intervention and infrapopliteal disease: Intraoperative evaluation of distal hemodynamic changes and foot blood volume of lower extremity

**DOI:** 10.3389/fsurg.2022.988639

**Published:** 2022-09-15

**Authors:** Chaonan Wang, Junye Chen, Jinsong Lei, Jiang Shao, Zhichao Lai, Kang Li, Wenteng Cao, Xiaolong Liu, Jinghui Yuan, Bao Liu

**Affiliations:** ^1^ Department of Vascular Surgery, Peking Union Medical College Hospital, Peking Union Medical College, Chinese Academy of Medical Sciences, Beijing, China; ^2^ Eight-year Program of Clinical Medicine, Peking Union Medical College Hospital, Peking Union Medical College, Chinese Academy of Medical Sciences, Beijing, China

**Keywords:** peripheral arterial disease, 2D perfusion angiography, blood volume, perfusion imaging, angiosome word count: 3291 angiosome-directed endovascular intervention

## Abstract

**Objectives:**

To evaluate foot blood volume and hemodynamics and explore whether quantitative techniques can guide revascularization.

**Materials and methods:**

A prospective single-center cohort study included thirty-three patients with infrapopliteal artery occlusion who underwent percutaneous transluminal angioplasty (PTA) between November 2016 and May 2020. The time-to-peak (TTP) from color-coded quantitative digital subtraction angiography (CCQ-DSA) and parenchymal blood volume (PBV) were used to evaluate the blood volume and hemodynamic changes in different regions of the foot before and after the operation.

**Results:**

After the intervention procedure, the overall blood volume significantly increased from 25.15 ± 21.1 ml/1,000 ml to 72.33 ± 29.3 ml/1,000 ml (*p* < 0.001, with an average increase of 47.18 ml/1,000 ml. The overall TTP decrease rate, postoperative blood flow time significantly faster than those preoperatively, from 22.93 ± 7.83 to 14.85 ± 5.9 s (*p* < 0.001, with an average decrease of 8.08 s). Direct revascularization (DR) resulted in significant blood volume improvement than compared with indirect revascularization (IR) [188% (28, 320) vs.51% (10, 110), *p* = 0.029]. Patients with DR had a significantly faster blood flow time than those with IR [80% (12, 180) vs. 26% (5, 80), *p* = 0.032]. The ankle-brachial index (ABI) of the affected extremity also showed an significant change from 0.49 ± 0.3 to 0.63 ± 0.24 (*p* < 0.001) after the intervention. The relative values of ΔTTP and ΔABI showed a weak correlation (*r* = −0.330).

**Conclusions:**

The quantitative measurement results based on PBV and CCQ-DSA techniques showed that the overall blood volume increased significantly and that the foot distal hemodynamics were significantly improved after endovascular treatment. DR in the ischemic area could r improve foot perfusion.

## Introduction

Infrapopliteal artery occlusive disease (IPOD) is one of the major causes of critical limb threatening ischemia (CLTI). Currently, the global prevalence of IPOD is between 4.5% and 29%, and the majority of patients live in low- and middle-income countries (1). CLTI has a poor prognosis and is often accompanied by diabetic foot ulcers. Studies have shown that without treatment, the amputation rate of CLTI patients within one year is approximately 27%, and the 5-year mortality rate is approximately 46% (2). In recent years, percutaneous transarterial angioplasty has gradually become the primary treatment for CLTI (3). The recovery of tissue lost to CLTI is closely related to the microcirculation of the foot.

In angiosome theory, the epidermis and subcutaneous tissue are supplied by specific blood vessels in the lower extremities (4). Each part is supplied by an independent artery, and the blood communication between them is limited. When the supplying artery of a specific area is occluded, clinical symptoms will appear in the corresponding area. Direct revascularization (DR) of the occluded artery can directly improve the tissue perfusion of the corresponding area. The curative effect is better than indirect revascularization (IR) (5, 6). Therefore, a more accurate assessment of the perfusion of the lower limbs could guide revascularization of the affected vessels and improve foot microcirculation, which play important roles in the recovery of lost tissue and the long-term prognosis (7, 8). However, the existing available research on the clinical practice of this theory is still controversial.

Currently, the traditional methods of perfusion assessment of IPOD include magnetic resonance angiography (MRA), computed tomographic angiography (CTA), and TcPO2 (9). Although DSA and CTA provide spatial resolution and contrast sensitivity levels that should be adequate to measure blood levels in arteries, they lack the temporal resolution and quantitative capabilities necessary to assess changes in the blood flow and volume in target regions (10). There is another obvious problem with the above techniques: lack of quantification.

An angiographic imaging system based on flat panel detector CT (FD-CT) can reconstruct the perfusion status in real time to generate an estimate of the parenchymal blood volume (PBV) (11, 12). Intraoperative PBV technology has been successfully used to evaluate the perfusion of other organs, and preliminary explorations have been made in the field of infrapopliteal endovascular interventional therapy (13–15). So far, 2D perfusion angiography technology has been used to evaluate the perfusion status and microcirculation in CLTI patients and further quantify the perfusion of lower extremity arterial tissue (16–19). TTP measured by color-coded quantitative digital subtraction angiography (CCQ-DSA) can assess hemodynamic changes after interventional treatment for peripheral arterial disease (PAD) of the lower extremities (20). Through intraoperative DSA scanning, quantitative color-coded blood flow maps and tissue perfusion maps are derived from the DSA datasets with a postprocessing system. Blood flow, contrast medium (CM) density, PBV and other information are displayed in real time, making percutaneous transarterial angioplasty (PTA) treatment more accurate and objective. To date, PBV has not been used with CCQ-DSA in IPOD treatment for the quantitative assessment of intraoperative perfusion.

Therefore, the purpose of this study was to evaluate the feasibility of PBV and CCQ-DSA in measuring the blood volume and hemodynamics of different foot angiosomes in patients with IPOD during treatment, and to explore the clinical correlation between technical methods and perioperative quantitative parameters, to provide a basis for selecting appropriate diseased arteries for treatment.

## Materials and methods

### Patient population

This study was a single-center prospective observational cohort study that was approved by the the ethics committee of Peking Union Medical College Hospital (JS-1037) and registered on Clinical Trials (NCT03248323). We recruited patients with inferior arterial occlusive disease (IPOD) from November 2016 to May 2020. The inclusion criteria were infrapopliteal artery stenosis or occlusion, indicated by duplex or CTA, and lower limb ischemia above Rutherford stage III. There was no heavy stenosis in the SFA (stenosis <30%) or short lesion length (length ≤5 cm, stenosis ≥30%). All patients' ankle-brachial indices (ABIs) were <0.9 on the target limbs. The exclusion criteria were an allergy to iodinated contrast medium, renal impairment, pregnancy and severe metabolic disease. Signed informed consent was obtained from all patients, and the clinical records for all patients were collected and reviewed.

### ABI measurement and evaluation

All patients rested supine in a room with a suitable temperature for at least 5–10 min. Blood pressure cuffs were placed on both arms and ankles, and then the systolic blood pressure of the antecubital fossae of both arms, posterior tibial arteries of both legs and dorsalis pedis arteries was recorded. Higher brachial artery pressures and dorsalis pedis or posterior tibial artery pressures were used for the index. ABI was measured 1–3 days before interventional treatment and 1 week after treatment. An ABI of 0.90 or less indicates abnormal PAD. Increasing ABI up to ≥0.15 after endovascular treatment indicated improved arterial flow (21, 22).

### Surgical procedure

During the procedure, each patient underwent compound anesthesia, and all were injected with 75 IU/kg heparin after intubation. Most occlusions were treated by an intraluminal approach, while a subintimal method was used when necessary, depending on the operator's judgment. DSA was first performed to clearly localize the lesion, and the lesion was treated with either plain balloons or drug-coated balloons (DCBs) using different balloons to enlarge the occluded segment. Additional DSA verified patency of the occluded artery. After completion of the procedure, the 6F sheath was removed, and hemostasis was performed with a vascular closure device.

### Image acquisition and analysis

All enrolled patients were treated with percutaneous transarterial angioplasty, with CCQ-DSA images and PBV image datasets collected by DSA series during the operation. Bilateral ABIs were collected before and after the operation.

**Figure 1:**
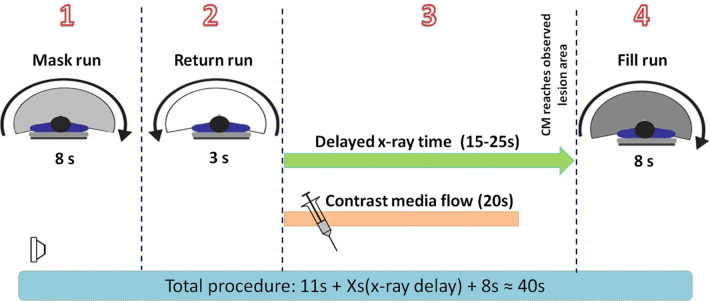
Time scale for the entire image acquisition. (1) An 8-s mask run without contrast medium injection was performed. (2) It took 3 s for the C-arm to return to the start position. (3) The contrast medium was injected automatically with a power injector, and the FD-CT system entered the delayed X-ray time period. The delay time ranged from 20 to 25 s for the preoperative procedure and from 15 to 20 s for the postoperative procedure. (4) An 8-s fill run with contrast medium injected was triggered automatically when the delayed X-ray time ran out. At this time, it was predicted that the CM had already reached the lesion and saturated the tissue with perfusion, thus providing the most accurate image of the hemodynamic status. CM, contrast medium.

Procedures were performed in a hybrid operating room. Dedicated CCQ-DSA images and PBV acquisition were performed using an angiographic system (Artis Zeego; Siemens, Forchheim, Germany). The system consisted of an 8-s 3D DSA protocol and allowed the automatic transfer of acquired images to the postprocessing workstation (syngo® X-Workplace, Siemens Healthcare GmbH, Forchheim, Germany) (Figure 1). CCQ-DSA images and PBV map acquisition were performed pre- and postoperatively during the endovascular procedure. The datasets were automatically transferred to the dedicated workstation for postprocessing *via* commercially available software, syngo iFlow (syngo® iFlow, Siemens AG Health care Sector, Germany) and syngo Dyna PBV (syngo® Dyna PBV, Siemens AG Health care Sector, Germany). The parameters for the CT scan were as follows: tube voltage 70 kV; acquisition time 8 s; flat Panel 12 detector size 30 cm × 40 cm; matrix 616 × 480; rotation angle 200°; 0.5°/frame; dose 0.36 13 µGy/frame. The scan area contained ankle and foot. Whole perfusion imaging (FD-PBV) was performed using the 8-sDSA program (Neuro PBV IR, Siemens AG Health care Sector, Forchheim, Germany). The effective dose value received by patients brain undergoing program was approximately 2.3 mSv (8 s) (23).The effective exposure time 16 s.

**Figure 2:**
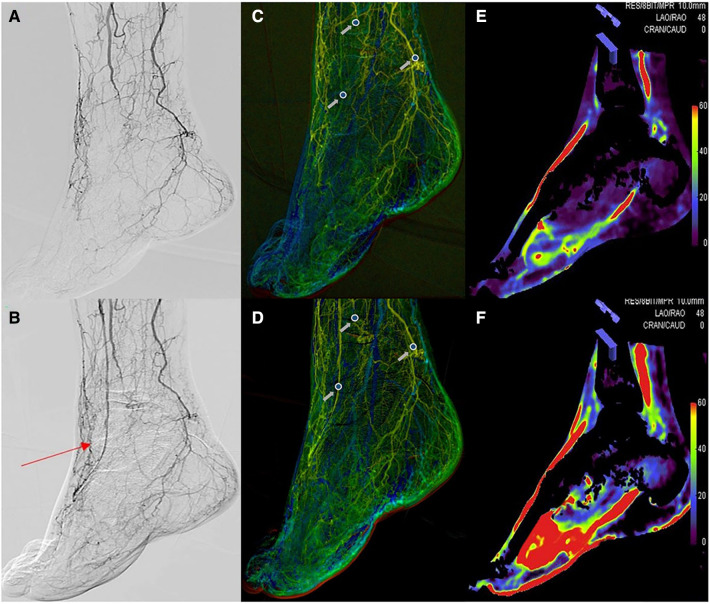
The situation before (**A**) and after (**B**) dorsal artery stenosis of the endovascular intervention. Region of interest measurements on color-coded quantitative digital subtraction angiography images before (**C**) and after (**D**) the endovascular procedure. The blood volume maps were visualized as colored perfusion maps before (**E**) and after (**F**) the endovascular procedure. The red arrow shows the dorsalis pedis artery after endovascular treatment. The gray arrow represents the pre- and postoperative time-to-peak at the dorsalis pedis artery, peroneal artery, and posterior tibial artery.

CCQ-DSA images (syngo® iFlow; Siemens) converted a two dimensional (2D)-DSA series into one color map, reflecting the progress of the contrast intensity changes over time. In the color-coded map, the delay time from contrast medium injection to the maximum intensity is calculated for each pixel and then is color-coded from the red (early maximum intensity) to blue (late maximum intensity) (15). Pre- and postoperative series were converted into two color-coded maps, in which regions of interest (ROIs) were selected within the vascular structure, and time-density curves (TDCs) and time-to-peak (TTP) were automatically computed, which served as parameters indicating the real-time hemodynamic status of the foot. The selection of the ROIs was made in accordance with the angiosome concept. We chose the proximal SFA as a reference ROI, and TTP_sfa_ represents its hemodynamic status. Other ROIs were placed in the three different regions of the dorsum, plantar surface, lateral malleolus, and the TTP_dor_, TTP_pla_, and TTP_lat_ were used to represent the hemodynamic status of the anterior tibial artery (ATA), posterior tibial artery (PTA) and peroneal artery (PA), respectively. To assess the distal hemodynamic improvement, we used the average distal hemodynamic improvement of 3 selected ROIs to indicate the hemodynamic status of the area. To reduce the influence of the individual's blood flow, the overall hemodynamic improvement needs to subtract TTP_sfa_ from the average hemodynamic improvement of TTP_dor_, TTP_pla_, and TTP_lat_ to obtain TTP_overall_. It should be noted that the catheter was placed at the same site throughout the whole DSA acquisition to liminate any disturbances of catheter movement on the TTP acquisition, so we could presume that the TTPs pre- and postoperation were acquired at the same baseline (Figure 2).

**Figure 3:**
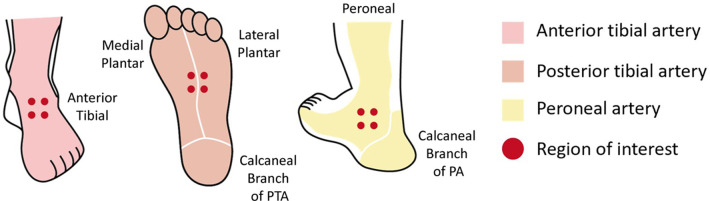
Six angiosomes of the foot supplied by the ATA, PTA, and PA are represented by different colors. Dots indicate the ROI. ATA, anterior tibial artery; PTA, posterior tibial artery; PA, peroneal artery; ROI, region of interest.

PBV acquisition consists of two 8 s 3D-DSA protocols, a mask run and a fill run. Before contrast medium injection, an 8 s mask run was performed, and then 30 ml of diluted contrast medium (CM, Visipaque 320 mg I/mL; GE Healthcare) (CM 15 ml + saline 15 ml = total volume, 30 ml) was injected through a catheter at a rate of 1.5 ml/s. The delay time of the X-ray for the fill run depended on the distal ROI's TDC generated from iFlow. When the TDC was in a steady state (almost the beginning of TTP), the second 8 s rotational fill run was performed. Data were then transferred to the workstation for 3D volume reconstructions and colored multiplanar reconstruction in which the blood volume of the tissue was color coded. Corrections were made to ensure that the preoperative and postoperative reconstructions stayed in the same position. Then, planes with a thickness of 0.5 mm were used to obtain a color-coded perfusion map of the intersecting area. The blood volume in the cylinder was calculated after selecting ROIs in the perfusion map. The ROI decision was also based on the concept of angiosomes. For the division of the perfusion regions in the foot and ankle corresponding to different vessels, three regions were selected for blood volume measurement for each PBV acquisition image, including the dorsum of the foot (corresponding to the ATA blood supply), the plantar surface (corresponding to the PTA blood supply) and the lateral malleolus (corresponding to the PA blood supply), and the measurements were defined as PBV_dor_, PBV_pla_ and PBV_lat_, respectively. To calculate the PBV of each region, we selected four ROIs with a diameter of 5 mm and used the average blood volume of the four ROIs to represent the blood volume of the region. The ROIs were positioned in the same place in the preoperative and postoperative perfusion maps. The blood volume of the entire foot was obtained by calculating the average of PBVdor, PBVpla, and PBV_lat_ and was expressed as PBV_overall_. The person who measured the blood volume was blinded to the clinical condition of the patients before measurement (Figure 3).

In addition, to assess whether direct revascularization (DR) of the target vessel in the ischemic area is better than indirect revascularization (IR) of the vessels in the adjacent area, we further grouped the measured areas in all patients, and if the vessels supplying a particular area had lesions and were DR, the region supplied by this artery would be sorted into the DR group; if the vessels supplying a specific area had lesions but were not DR, and the vessels supplying the adjacent angiosome was revascularized to achieve indirect blood supply, the region was classified as the IR group; if no lesions were present in the supplying artery, the corresponding region was sorted into the patent group (PG), whose blood volume change was used as a blank contrast.

### Statistical analysis

Baseline characteristics are expressed as frequencies and percentages. The Shapiro-Wilk test was used to determine the normality of the continuous variable distributions. Paired t tests were used to examine the differences between the preoperative and postoperative blood volume, TTP, and ABI and Cr(E). The Wilcoxon signed-rank test was used to assess differences in the blood volume distributions between the DR, IR, and PG groups, and the median (quartiles) was used to describe the data. A *p* value ≤0.05 was considered statistically significant. Pearson's correlation coefficient *p* was calculated among TTP in CCQ-DSA and ABI of the affected extremity. Statistical analysis was conducted using SPSS version 25 (SPSS Inc).

## Results

### Patients

The epidemiologic characteristics of the patients are listed in Table 1. A total of 99 arteries from 33 patients were considered for analysis. Of these, 15 arteries were patent and 84 arteries had stenosis or occlusion, including 31 ATA, 23 PTA, and 30 PA. Of these 84 diseased arteries, 40 arteries received endovascular intervention, including 15 ATA, 9 PTA, and 16 PA, and 11 arteries were treated with drug-eluting balloons without stenting. The patients were followed up one month after the intervention to evaluate their clinical condition.

**Table 1:** Demography of study population.

Patients no.	33
Average age (range)	66.15 (46–86)
Males, % (ratio)	72.73 (24/33)
Comorbidities, % (ratio)	
Hypertension	69.70 (23/33)
Diabetes mellitus	78.79 (26/33)
Coronary artery disease	36.36 (12/33)
Hyperlipidemia	30.30 (10/33)
Cerebral infarction	21.21 (7/33)
Smoking	21.21 (7/33)
Mild renal dysfunction	12.12 (4/33)
Ankle-brachial index	0.41 ± 0.12
Main lesion position, % (ratio)	
Anterior tibial artery	45.45 (15/33)
Posterior tibial artery	27.27 (9/33)
Peroneal artery	48.48 (16/33)
Preoperative Rutherford stage, % (ratio)	
4	66.67 (22/33)
5	18.18 (6/33)
6	15.15 (5/33)
Postoperative Rutherford stage, % (ratio)	
<3	9.09 (3/33)
3	48.48 (16/33)
4	15.15 (5/33)
5	9.09 (3/33)
6	18.18 (6/33)

### Quantitative analysis for perfusion assessment

We compared the overall perfusion condition before and after the endovascular procedure. The preoperative blood volume and the postoperative blood volume were significantly different between these 2 groups (25.15 ± 21.1 ml/1,000 ml vs. 72.33 ± 29.3 ml/1,000 ml, *p* < 0.001). After the operation, the creatinine (Cr) level did not change significantly. The total Cr (µmol/L) showed no considerable changes from 85.27 ± 32.13 to 83.39 ± 32 (*p* = 0.151), with an MD of −1.88 (Table 2).

**Table 2:** TTP values, Overall blood volume, ABI and Cr (E) values preoperative and postoperative.

Parameter	Pre-operation	Post-operation	*p*	MD
	Mean ± SD	Mean ± SD		
TTP (s)	22.93 ± 7.83	11.85 ± 5.9	*p *< 0.001	−11.08
Blood volume (ml)	25.15 ± 21.1	72.33 ± 29.3	*p *< 0.001	47.18
ABI	0.49 ± 0.3	0.63 ± 0.24	*p *= 0.0005	0.14
Cr(E) (µmol/L)	85.27 ± 32.13	83.39 ± 32	*p *= 0.1512	−1.88

Normal range of Cr(E), 59–104 μmol/L. MD, mean difference, *p *≤ 0.05; TTP, time-to-peak; ABI, ankle brachial index.

**Figure 4:**
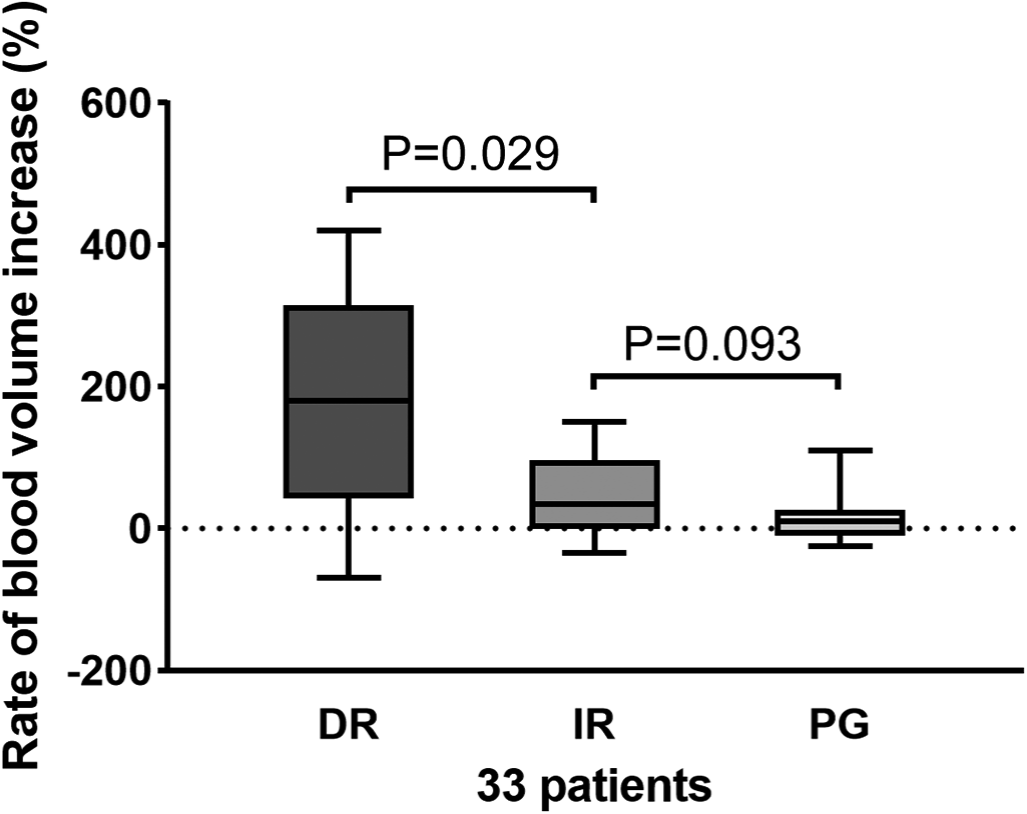
Increase rate of blood volume in the DR, IR, and PG groups. A significant difference was found between the DR and IR groups (*p* = 0.029), while there was no difference between the IR and PG groups (*p* = 0.093). DR, direct revascularization; IR, indirect revascularization; PG, patent group.

Then, we further grouped the perfusion data and analyzed the increase in blood volume in the DR, IR and PG groups. The PG group contained 15 regions, the DR group contained 40 regions, and the IR group contained 44 regions. After endovascular intervention, DR resulted in significant blood volume improvement compared with IR [188% (28, 320) vs. 51% (10, 110), *p* = 0.029], respectively. However, there was no difference between the IR and PR groups in the blood volume rate [51% (10, 110) vs. 32% (−15, 32), *p* = 0.093]. These results show that direct revascularization according to the location of the lesion can improve the blood volume (Figure 4).

### ABI and hemodynamic changes measurement

**Figure 5:**
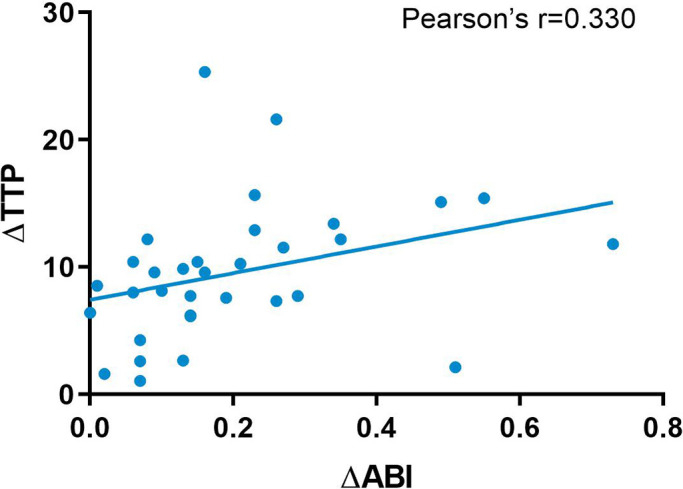
Pearson correlation analysis between TTP and ABI. TTP, time-to-peak; ABI, ankle-brachial index.

We used the ABI index and CCQ-DSA to evaluate distal hemodynamic changes and their correlation. The TTP overall decreased significantly from 22.93 ± 7.83 to 14.85 ± 5.9 (*p* < 0.001), with an average decrease of 8.08 s. The ABI of the affected extremity also showed an obvious increase from 0.49 ± 0.3 to 0.63 ± 0.24 (*p* < 0.001). The mean difference (MD) of ABI was 0.14 (Table 2). The Pearson correlation coefficient was applied between the relative values of ΔABI and ΔTTP. ΔTTP/ΔABI showed a weak correlation (*r* = 0.330) (ΔABI indicated the an increase in ABI after revascularization, ΔABI = ABI post – ABI pre; ΔTTP indicated a decrease in time-to-peak after revascularization, ΔTTP = TTP pre – TTP post) (Figure 5).

**Figure 6:**
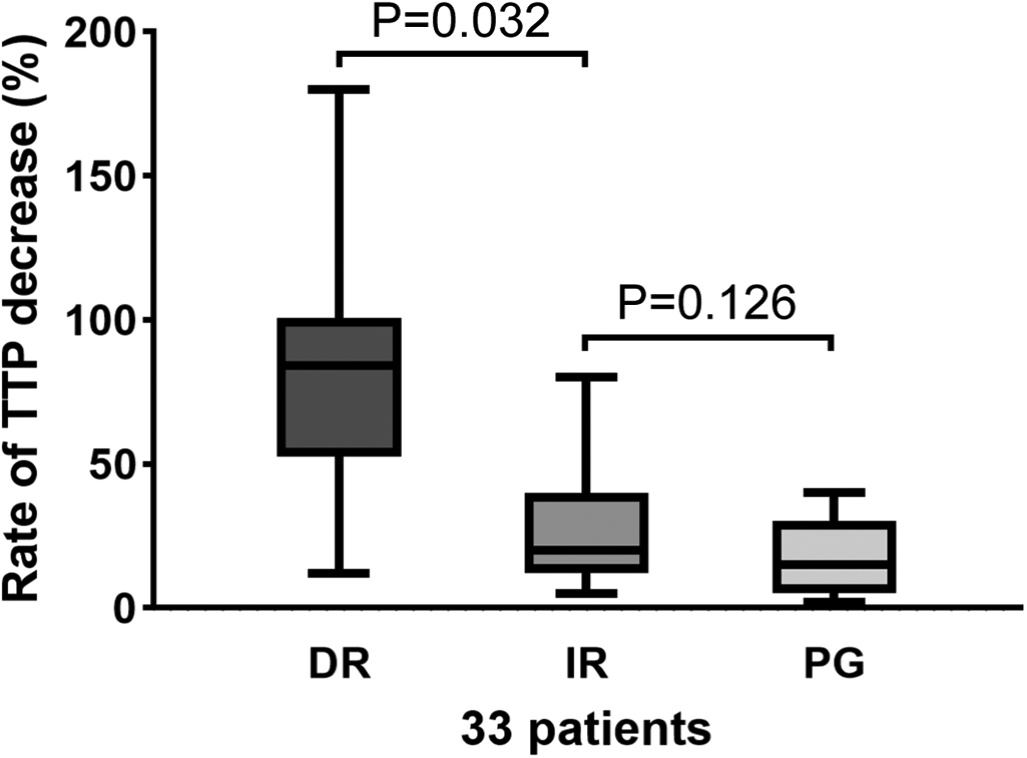
TTP decreased the rate in the DR, IR, and PG groups. There was a significant difference between the DR and IR groups (*p* = 0.032). There was no significant change in the IR and PG groups (*p* = 0.126). DR, direct revascularization; IR, indirect revascularization; PG, patent group; TTP, time-to-peak.

In patients with a decreased TTP rate, DR resulted in a significantly faster blood flow time than IR [80% (12, 180) vs. 26% (5, 80), *p* = 0.032]. For the IR and PR groups, no difference was revealed [26 (5, 80) vs. 17(2, 44), *p* = 0.126] (Figure 6).

### One-month follow-up results

At the one-month follow-up, among the 22 patients with Rutherford stage 4, 19 patients had reduced to stage 3 and below; seventeen patients had intermittent claudication symptoms lower than stage 4; and the remaining two patients had complete remission of clinical symptoms. However, three patients remained at stage 4. Among the last 11 patients with stage 5/6 disease, two patients had healed foot wounds; three people showed healing progress, with smaller ulceration areas by the naked eye; and,, six people remained the same with unhealed ulcers, and further follow-up is needed for recovery of the ulcers. One patient had more severe symptoms that increased to grade 6.

## Discussion

In this study, for the first time, PBV and CCQ-DSA were used in endovascular intervention procedures to evaluate the patient's intraoperative perfusion and hemodynamics, thereby overcoming some deficiencies of the currently available imaging techniques.

Some studies support the application of the concept of angiosomes to guide revascularization (24, 25). This concept should also be considered when revascularizing diseased infrapopliteal arteries, and the ESC guidelines have also recommended it (26). Compared with IR, angiosome-targeted DR results in a significant increase in wound healing and blood flow (27). The direct blood flow obtained from DR based on the angiosome model is more beneficial to foot wound healing in patients with diabetes (28). However, this concept is still in dispute. In vascular disease, tissue oxygen and nutritional deficiency can be overcome through the formation of collateral circulation and changes in microcirculation (29). Therefore, the concept of the angiosome may not correspond to the lesion-related artery in patients with CLTI. A study has shown that pedal arch quality is directly related to wound healing and does not follow the revascularized angiosome (30). Some scholars believe that the results of existing studies show that the classical angiosome concept is not applicable to peripheral arterial diseases.

At the same time, some studies have also observed that DR and IR affect the perfusion state between different angiosomes. Patients who receive tibial artery revascularization have different microcirculation states on the dorsal side and the plantar side (31). Intraoperative fluorescein angiography was used to compare skin microcirculation differences between the DR and IR angiosomes (32). Some scholars also use laser Doppler flowmetry and tissue spectrometry to evaluate the impact of different reconstruction methods on skin microcirculation. However, while the skin perfusion was significantly improved after the interventions, but DR and IR showed no differences in these studies. Due to the limitations of these 2D evaluation methods, the accuracy of the results is affected. Some scholars also use CT, MRI and SPECT/CT perfusion technology to assess the foot blood volume, and based on angiosomes, the perfusion of the foot was evaluated, and preliminary results were obtained (33–35). Our results showed that DR of the ischemic area has better perfusion improvement than the establishment of revascularization through IR (the collateral circulation). This may provide a new quantitative basis for doctors to develop more effective revascularization strategies.

In addition, we discussed the changes in hemodynamics in the DR and IR groups. We found that the TTP of the DR group was significantly lower than that of the IR group. ΔTTP can be regarded as an index of changes in the circulation of the distal foot and arterial patency. In this study, we found a weak correlation between the TTP change ratio and the ABI index. ABI measurement, as the gold standard of IPAOD diagnosis, showed a significant improvement after PTA and is widely used to monitor the efficacy of revascularization (36). However, ABI is usually unreliable in patients with diabetic foot due to severe arterial calcification (37). According to our results, CCQ-DSA technology is expected to become a quantitative indicator that can evaluate the improvement of the foot circulation and hemodynamic changes. Therefore, it could be combined with the ABI index or works as an alternative measurement tool in cases of ABI measurement failure, skin damage, and severe calcification.

We did not analyze the correlation between clinical outcomes, that is, the Rutherford classification and the changes in blood volume and hemodynamics, mainly due to the difference in the collateral circulation among individuals and the effect of diabetic neuropathy on clinical symptoms. There is a theory that the quality of the pedal arch is related to the clinical outcomes after revascularization (31), and the patency of the peroneal artery and pedal arch are directly related to the clinical outcomes of patients (38).

The present study has several limitations that should be considered. First, the sample size was too small and this was only a prospective observational cohort study, and randomized controlled trials should be conducted in the future to obtain better and higher quality clinical evidence. Second, although fusion was performed for the pre- and postoperative reconstructions, foot deformation and movement during the treatment could not be eliminated entirely.

## Conclusion

In the present study, we used the PBV and CCQ-DSA techniques quantitatively to evaluate blood volume and hemodynamics during endovascular treatment. Our findings show that overall blood volume increased significantly and distal hemodynamic improvement occurred in the foot after endovascular treatment. During the process of intervention, according to the theory of angiosomes, revascularizing directly in the ischemic area can improve foot perfusion better than relying solely on the collateral circulation, which was directly confirmed by the changes in blood volume and hemodynamics.

## Data Availability

'The original contributions presented in the study are included in the article/supplementary materials, further inquiries can be directed to the corresponding author/s.
